# Clinical Outcomes of Ultrasound-Guided IPACK Block for Posterior Knee Pain in Advanced Osteoarthritis: A Multicenter Prospective Single-Arm Study

**DOI:** 10.3390/jcm15166410

**Published:** 2026-08-19

**Authors:** Ridvan Isik, Onur Balaban, Muhammed Zahid Sahin, Ali Eman, Emre Uzun, Gul Devrimsel, Kemal Nas

**Affiliations:** 1 Department of Physical Medicine and Rehabilitation, Division of Pain Medicine, Sakarya Training and Research Hospital, 54290 Sakarya, Turkey; dr.ridvanisik@gmail.com; 2 Department of Anesthesiology and Reanimation, Faculty of Medicine, Sakarya University, 54290 Sakarya, Turkey; obalabandr@gmail.com; 3 Department of Physical Medicine and Rehabilitation, Faculty of Medicine, Sakarya University, 54290 Sakarya, Turkey; 4 Department of Anesthesiology and Reanimation, Sakarya Training and Research Hospital, 54290 Sakarya, Turkey; dralieman02@gmail.com; 5 Department of Physical Medicine and Rehabilitation, Sakarya Training and Research Hospital, 54290 Sakarya, Turkey; ftremreuzun@gmail.com; 6 Department of Physical Medicine and Rehabilitation, Faculty of Medicine, Recep Tayyip Erdoğan University, 53100 Rize, Turkey; gul.devrimsel@erdogan.edu.tr; 7 Department of Physical Medicine and Rehabilitation, Division of Pain Medicine, Faculty of Medicine, Sakarya University, 54290 Sakarya, Turkey; kemalnas@sakarya.edu.tr

**Keywords:** knee osteoarthritis, posterior knee pain, IPACK block, ultrasound-guided nerve block, pain management

## Abstract

**Background/Objectives**: Posterior knee pain is a common but often overlooked component of knee osteoarthritis (KOA), especially in advanced stages. Conventional nerve blocks targeting anterior innervation may provide insufficient relief. The IPACK block selectively targets posterior capsular innervation; however, evidence for its effectiveness in chronic KOA-related posterior pain remains limited. **Methods**: In this multicenter, prospective, single-arm, nonrandomized interventional study, patients aged 50–90 years with Kellgren–Lawrence grade III-IV KOA and predominant posterior knee pain unresponsive to conservative treatment received an ultrasound-guided IPACK block. Pain (NRS), function (WOMAC), and quality of life (SF-12) were assessed at baseline, 1 month, and 3 months. **Results**: Eighty-four patients (84 knees) were included. Mean NRS decreased from 7.8 ± 1.3 to 3.5 ± 1.5 at 1 month and 6.7 ± 1.3 at 3 months (*p* < 0.001). WOMAC improved from 71.5 ± 11.0 to 37.3 ± 10.4 and 60.2 ± 10.5 at 1 and 3 months, respectively (*p* < 0.001). SF-12 physical and mental scores also improved significantly (*p* < 0.001). **Conclusions**: Improvements in pain, physical function, and health-related quality-of-life measures were observed during the three-month period following the IPACK block. No serious procedure-related adverse events were recorded during the three-month follow-up period.

## 1. Introduction

Knee osteoarthritis (KOA), characterized by chronic pain and cartilage deterioration, affects millions of people worldwide and is a leading cause of disability in older individuals. With the aging of the world population, its prevalence continues to increase, leading to significant morbidity and economic costs [1,2].

Pain distribution in knee osteoarthritis is heterogeneous and does not necessarily correspond directly to radiographic severity or a single anatomical compartment. Patients may describe medial or lateral joint-line pain, peripatellar or retropatellar pain, diffuse pain, or combinations of these patterns. Posterior knee pain is less frequently emphasized but may contribute to symptom burden in some patients with advanced disease [3].

Various treatment options exist, including conservative therapies, injection-based interventions, and surgical procedures [3]. In cases resistant to conservative treatments, interventional treatments or surgical options are required [4]. Injection techniques encompass intra-articular (such as steroids, hyaluronic acid, and ozone) and periarticular injections and sensory nerve blocks [4]. However, total knee arthroplasty remains the definitive treatment for appropriately selected patients with advanced symptomatic knee osteoarthritis after failure of nonoperative management [5].

Femoral, saphenous, or genicular nerve blocks are frequently employed for the relief of knee pain. These nerves supply anterior innervation to the knee; thus, procedures targeting them frequently do not achieve adequate analgesia in the posterior region of the knee [6,7]. Posterior knee pain can be caused by many factors, including knee osteoarthritis, posterior medial meniscus tear, strain in the hamstring and gastrocnemius muscles, and Baker’s cyst [8]. This issue is commonly observed in clinical practice, particularly among patients with advanced osteoarthritis, and is often neglected.

The IPACK (interspace between the popliteal artery and capsule of the posterior knee) block provides analgesia for posterior knee pain by blocking the sensory articular branches of the sciatic nerve [9]. This technique was initially employed to diminish post-operative pain and the necessity for analgesic medications following total knee arthroplasty [10,11]. Nevertheless, there is insufficient evidence concerning the effectiveness of IPACK blocks for posterior knee pain in individuals with knee osteoarthritis. In light of this research gap, the aim of this study was to evaluate changes in pain, physical function, and quality of life following the IPACK block in patients with posterior knee pain associated with knee osteoarthritis.

The primary outcome was the change in NRS pain scores from baseline to the one- and three-month follow-up assessments. Secondary outcomes were changes in functional capacity and quality of life at the one- and three-month follow-up assessments.

## 2. Materials and Methods

This study was prospectively registered at ClinicalTrials.gov. The trial is registered under the name “Knee Osteoarthritis and IPACK (IPACK)” with the registration number NCT06712394. Prospective registration was completed on 26 November 2024, and the first patient was enrolled on 1 January 2025. Although ethical approval was obtained earlier, participant enrollment commenced only after prospective trial registration had been completed. The full trial registration details are available at ClinicalTrials.gov (https://clinicaltrials.gov/study/NCT06712394#more-information accessed on 9 March 2024). This study was reported in accordance with the Strengthening the Reporting of Observational Studies in Epidemiology (STROBE) guidelines.

### 2.1. Study Design and Population

This multicenter, prospective, single-arm, nonrandomized interventional study was carried out at two university hospital outpatient pain clinics between January 2025 and May 2026. The study was approved by the Institutional Review Board of the Ethics Committee of Sakarya University (E-16214662-050.01.04-287837-131) and conducted in accordance with the principles of the Declaration of Helsinki. Written informed consent was obtained from all participants after they were thoroughly informed about the study’s objectives and potential risks.

Inclusion Criteria: Patients aged 50–90 years who were diagnosed with primary knee osteoarthritis according to the American College of Rheumatology [12] criteria, had Kellgren–Lawrence grade III and IV osteoarthritis, had posterior knee pain associated with this disease, had only a symptomatic knee, and did not respond to conservative treatments were included in the study.

Exclusion criteria: Patients with a history of any intra-articular injection within 3 months, a history of previous knee surgery, posterior knee pathologies such as meniscal tears, tendon or ligament injuries, Baker’s cyst, a diagnosis of lumbar radiculopathy, a diagnosis of malignancy, neuromuscular or coagulopathy disorders, missing follow-up parameters, a secondary knee osteoarthritis diagnosis (trauma, inflammatory joint disease, etc.) and systemic or local infection were excluded from the study.

**Figure 1:**
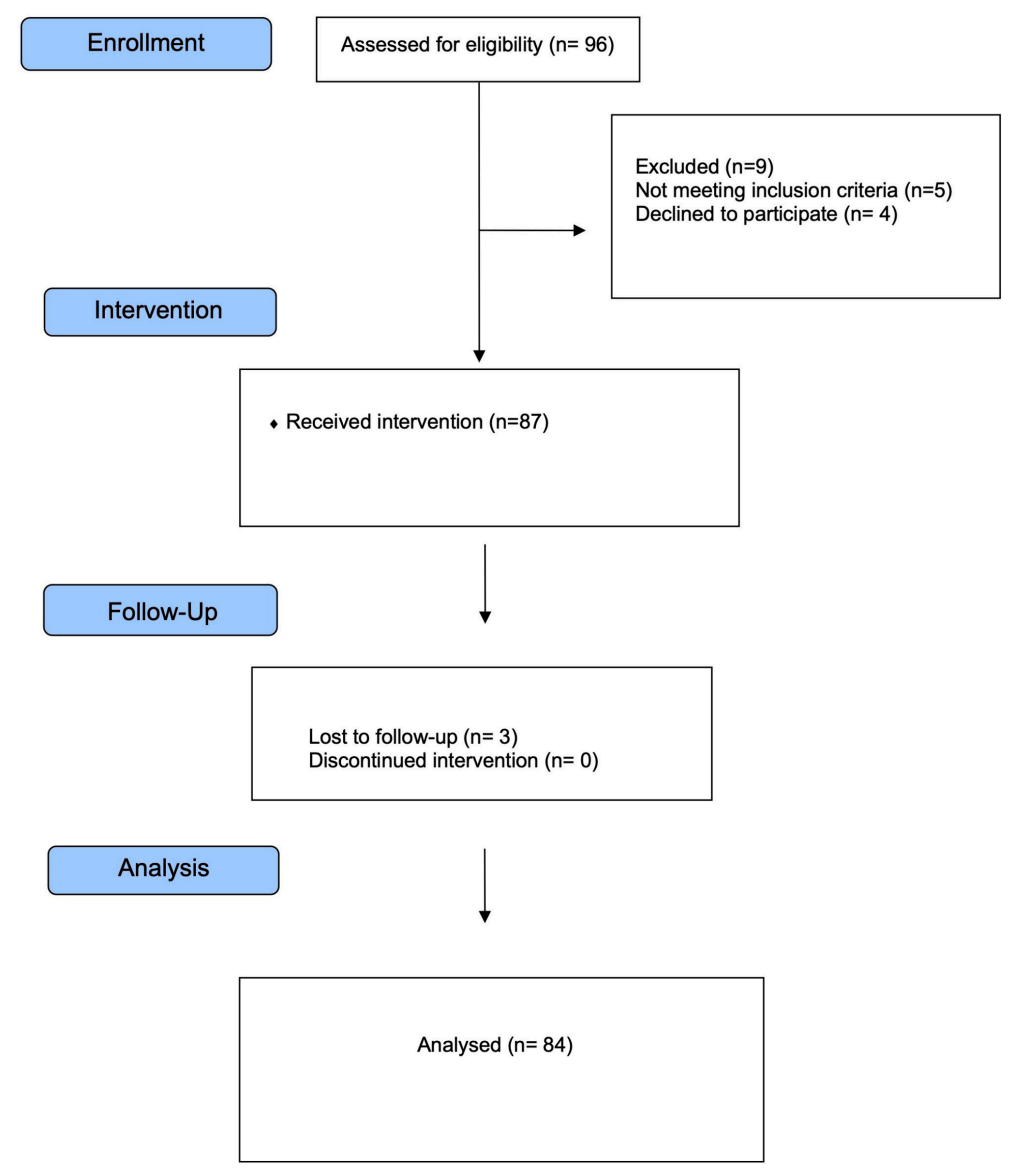
Patient selection flow chart.

The flowchart for determining the study population is displayed in Figure 1.

### 2.2. Injection Procedure

In both centers, we utilized ultrasound in all cases (HM70 EVO, Samsung Healthcare, Seoul, Republic of Korea) with a curvilinear ultrasound (US) transducer (3–16 MHz). The procedures were performed in the special block procedure room by two physicians experienced in ultrasound-guided pain interventions (R.I. and G.D.). Standard monitoring, including electrocardiography, non-invasive blood pressure, and peripheral oxygen saturation, was employed, and an IV catheter was inserted before the blocks.

**Figure 2:**
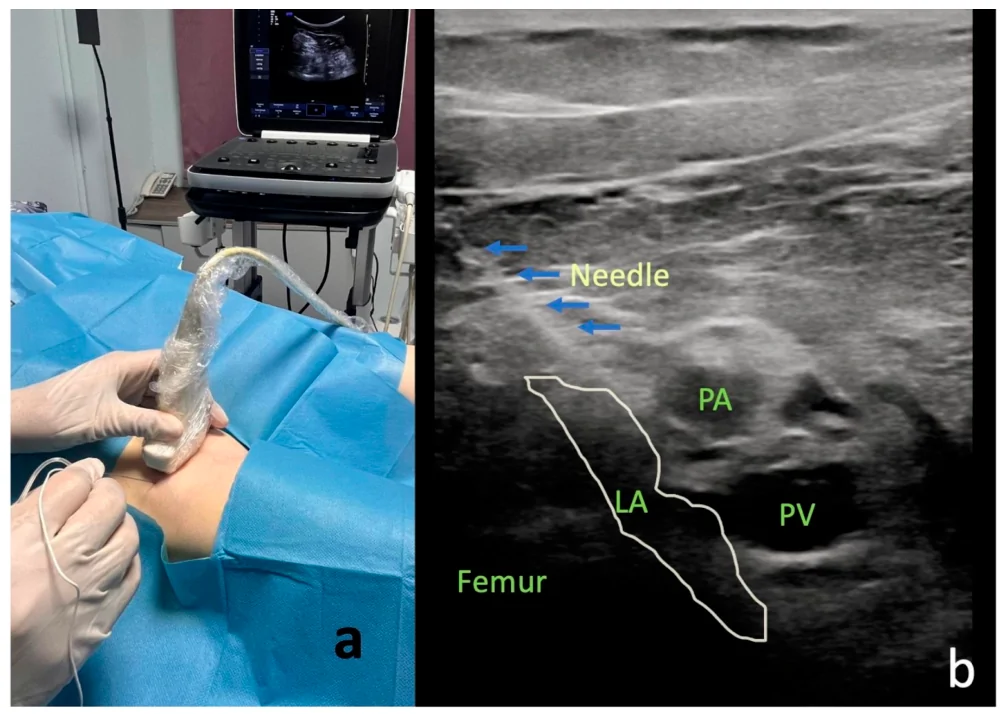
(**a**) Position of the ultrasound probe and needle during the IPACK block. The patient is in the prone position. The operator stands lateral to the patient with the ultrasound machine positioned in front of the operator. (**b**) Ultrasound image showing the needle trajectory, local anesthetic distribution, and relevant anatomical structures during the IPACK block. PA: popliteal artery, PV: popliteal vein, LA: local anesthetic.

The US transducer was enclosed in a sterile cover, and then the patient’s skin was sterilized with povidone–iodine. As the sonographic interface, a customized sterile gel was utilized. Patients were asked to lie prone, and the ultrasound probe was initially placed in a transverse plane (short axis) at the popliteal fossa in order to identify the popliteal vessels. Then, the probe was gradually moved distally to locate the space between the popliteal arteries and posterior aspects of the distal femoral shaft. A 22-gauge, 80 mm needle (Stimuplex A; B.Braun, Melsungen, Germany) was advanced from lateral to medial using an in-plane approach (Figure 2). Following the confirmation of negative aspiration, a total of 20 mL of a mixed solution, comprising 10 mL of 0.5% bupivacaine and 10 mL of saline, was administered, ensuring satisfactory fluid distribution in the region (Figure 2). Procedures were performed by two physicians experienced in ultrasound-guided pain interventions. Before study initiation, both operators agreed on a standardized protocol regarding patient positioning, ultrasound landmarks, needle trajectory, injectate composition and volume, and post-procedural monitoring.

All patients received a home exercise program consisting of quadriceps, hamstring, and hip muscle stretching and strengthening exercises. They were instructed to perform three sets of 10 repetitions daily until the one-month follow-up visit. All patients received the same home exercise recommendations; however, individual adherence, including exercise frequency, duration, and completion, was not systematically monitored or recorded.

During follow-up, patients were permitted to use NSAIDs or paracetamol as needed according to routine clinical practice. Analgesic treatment was not standardized by the study protocol, and patient-level data regarding the type, dose, frequency, duration, and cumulative amount of analgesic use were not systematically recorded. No additional interventional procedures were performed during the follow-up period.

Patients were monitored in the observation room for thirty minutes post-injection to assess for any potential complications. Patients without complications were discharged with recommendations and instructed to return for follow-up visits at 1 and 3 months.

### 2.3. Outcome Measures

The Numeric Rating Scale (NRS), the Western Ontario and McMaster Universities Arthritis Index (WOMAC), and the Short Form-12 Health Survey (SF-12) were assessed at baseline and at the one- and three-month follow-up visits. Demographic and clinical characteristics were recorded at baseline.

The NRS is a commonly employed measure for evaluating and monitoring pain intensity. It is an 11-point scale ranging from 0, indicating no pain, to 10, indicating the worst pain imaginable, and the patient is asked to rate his or her pain on the scale from 0 to 10 [13].

The WOMAC questionnaire was developed to assess joint function associated with osteoarthritis and consists of three main sections: pain, stiffness, and physical function. A Turkish validity and reliability study was conducted [14].

The SF-12 consists of twelve items that evaluate eight different health domains, providing an evaluation of both physical and mental well-being. The SF-12 provides two summary scores: the mental component score (MCS-12) and the physical component score (PCS-12). A decrease in the questionnaire score indicates progression of disability. A Turkish validity and reliability study was conducted [15].

The validated Turkish-language versions of the WOMAC and SF-12 instruments were administered, and the NRS was also applied in Turkish.

Because of the single-arm study design, neither the participants nor the investigators involved in follow-up assessments were blinded to the intervention. The NRS, WOMAC, and SF-12 were completed as patient-reported outcome measures at each assessment.

### 2.4. Statistical Analysis

An a priori sample size calculation was performed using G*Power software (version 3.1; Heinrich Heine University Düsseldorf, Düsseldorf, Germany). Based on the primary outcome measure (NRS), assuming a moderate-to-large effect size (Cohen’s f = 0.30), a significance level (α) of 0.05, and a statistical power (1 − β) of 80%, the minimum required sample size was calculated as 64 participants. To compensate for potential dropouts and incomplete follow-up, at least 80 participants were planned for inclusion. A total of 84 patients completed the study, exceeding the calculated minimum sample size. Sample size estimation was performed according to the methodology described by Faul et al. [16].

Statistical analyses were performed using IBM SPSS Statistics for Windows, version 28.0 (IBM Corp., Armonk, NY, USA). Descriptive statistics are presented as mean ± standard deviation (SD) for continuous variables and frequency (percentage) for categorical variables. The normality of continuous variables was assessed using the Shapiro–Wilk test. Since repeated measurements were not normally distributed, overall comparisons across the three assessment time points were performed using the Friedman test. Pairwise comparisons were subsequently conducted using Bonferroni-adjusted Wilcoxon signed-rank tests. Effect sizes for pairwise comparisons were calculated as r = Z/√N according to Rosenthal and interpreted using Cohen’s conventional thresholds (0.10 = small, 0.30 = medium, and 0.50 = large effect). Mean differences with corresponding 95% confidence intervals (95% CIs) were calculated for baseline-to-follow-up comparisons. Clinical significance was additionally evaluated using predefined minimal clinically important difference (MCID) thresholds. Clinically meaningful improvement in pain was defined as at least a 50% reduction in NRS from baseline [17]. For WOMAC, clinically meaningful improvement was defined as a decrease of at least 10 points from baseline [18]. For the Short Form-12 (SF-12), clinically meaningful improvement was defined as an increase of at least 5 points in both the Physical Component Summary (PCS-12) and Mental Component Summary (MCS-12) scores compared with baseline values [19].

Changes in MCID responder rates between the 1- and 3-month follow-up visits were analyzed using McNemar’s test. A two-sided *p* value < 0.05 was considered statistically significant. The analyses were performed using a complete-case approach and included patients with available outcome data at baseline and at both the one- and three-month follow-up assessments. Patients with incomplete follow-up data were excluded, and no imputation of missing values was performed.

The complete-case and per-protocol populations were identical because all 84 patients with complete outcome data received the intervention as planned and completed both follow-up assessments. Therefore, a separate sensitivity analysis comparing these two populations was not applicable.

An exploratory per-center analysis was performed by comparing clinical outcome scores and changes from baseline between the two participating centers using the Mann–Whitney U test. To account for multiple testing, Holm adjustment was applied across the eight between-center change-score comparisons.

## 3. Results

A total of 96 patients were assessed for eligibility. Five patients did not meet the eligibility criteria, and four declined participation. Of the 87 patients enrolled, three had incomplete follow-up data. Therefore, 84 patients (84 knees) completed all assessments and were included in the complete-case/per-protocol analysis (Figure 1).

Of the patients, 54 were female (64.3%), and 30 were male (35.7%). The mean body mass index was 30.5 kg/m^2^ with a standard deviation of 2.5. The mean duration of symptoms was 26.4 ± 16.6 months. The patients’ characteristics and demographic values are presented in Table 1.

**Figure 3:**
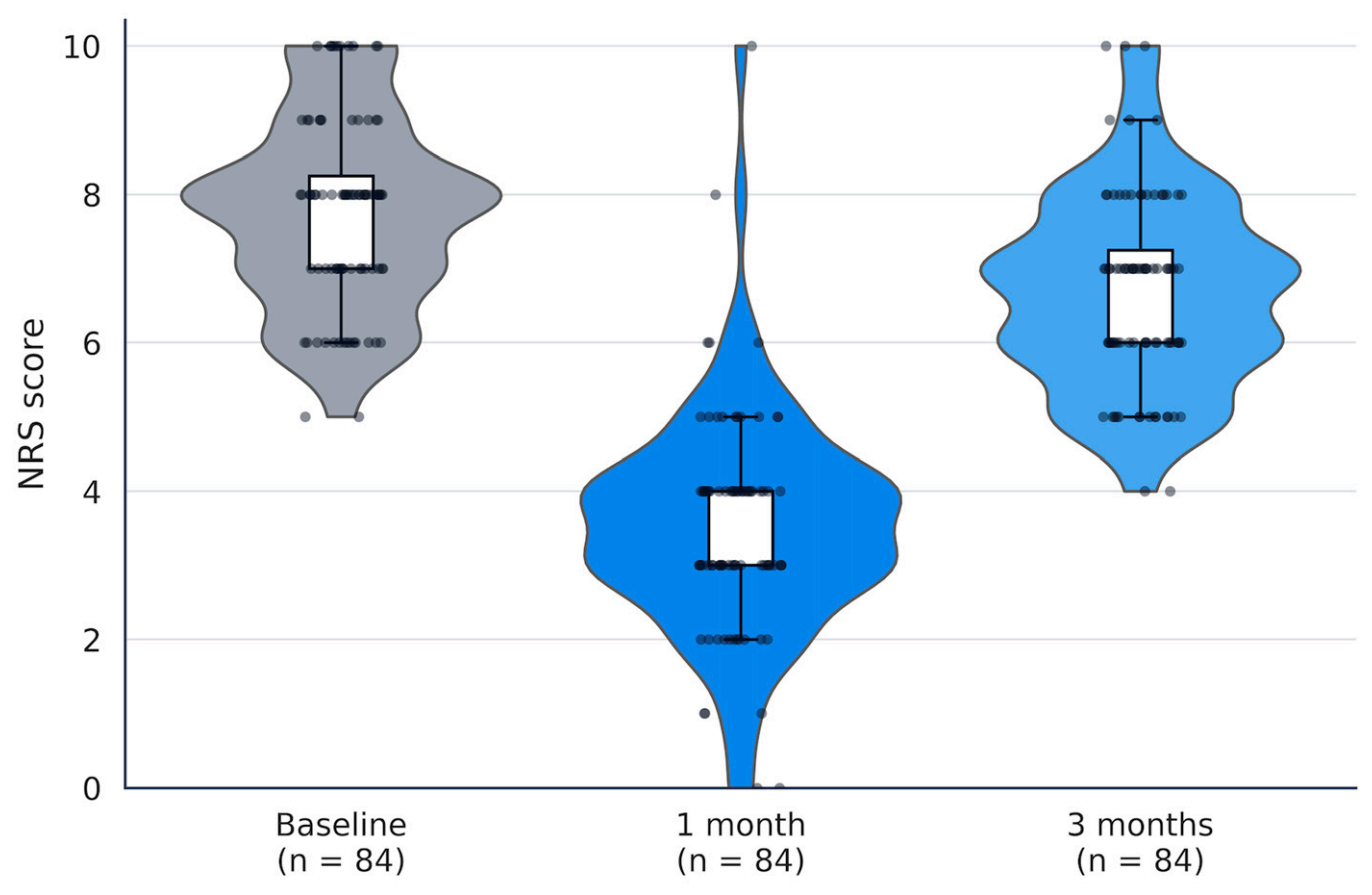
Distribution of NRS pain scores at baseline and at the 1- and 3-month follow-up assessments. Violin plots show the density distributions; boxes represent the interquartile range with median lines, and dots represent individual patients. Overall differences were assessed using the Friedman test (*p* < 0.001).

**Figure 4:**
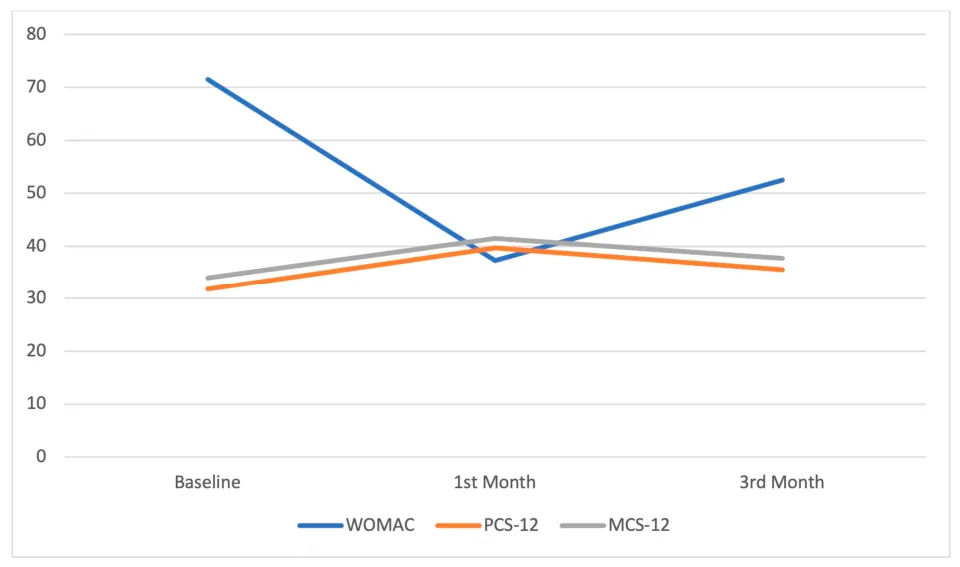
Mean WOMAC, PCS-12, and MCS-12 scores at baseline and during follow-up.

Pain intensity assessed by the NRS decreased significantly from baseline (7.8 ± 1.3) to 3.5 ± 1.5 after the first month and remained significantly lower than baseline after the third month (6.7 ± 1.3) (*p* < 0.001). Likewise, WOMAC scores demonstrated a marked improvement, decreasing from 71.5 ± 11.0 at baseline to 37.3 ± 10.4 after the first month and 60.2 ± 10.5 after the third month (*p* < 0.001). Although partial deterioration was observed between the first and third months, WOMAC scores remained significantly improved compared with baseline (Table 2, Figure 3 and Figure 4).

Health-related quality of life also improved significantly following treatment. PCS-12 scores increased from 31.8 ± 5.4 at baseline to 39.7 ± 5.5 after the first month and remained higher than baseline at 35.6 ± 5.3 after 3 months (*p* < 0.001). Similarly, MCS-12 scores increased from 34.0 ± 4.3 at baseline to 41.5 ± 4.9 after the first month and remained significantly improved at third month (37.7 ± 4.7; *p* < 0.001). Friedman analyses demonstrated significant overall differences across the three assessment time points for all outcome measures. Pairwise comparisons confirmed significant improvements between baseline and both follow-up assessments for all outcome measures after Bonferroni adjustment (all *p* < 0.001) (Table 2, Figure 3 and Figure 4).

Within-patient changes from baseline were associated with large effect-size estimates for all clinical outcome measures (Table 3). At the 1-month follow-up, the mean reduction in NRS score was 4.21 points (95% CI, 3.96–4.47), corresponding to a large effect size (r = 0.87). WOMAC scores improved by a mean of 34.17 points (95% CI, 31.99–36.34), while PCS-12 and MCS-12 increased by 7.84 (95% CI, 7.17–8.50) and 7.50 points (95% CI, 6.93–8.07), respectively; all comparisons demonstrated large effect sizes (r = 0.87).

At the 3-month follow-up, treatment effects within-patient changes remained statistically significant despite some attenuation compared with the 1-month evaluation. Baseline-to-3-month comparisons continued to demonstrate large effect sizes for NRS (r = 0.77), WOMAC (r = 0.85), PCS-12 (r = 0.87), and MCS-12 (r = 0.86), although the magnitude of the observed improvements had substantially attenuated by the three-month assessment (Table 3).

Clinical significance was additionally evaluated using predefined MCID responder thresholds (Table 4). At the 1-month follow-up, 65 of 84 patients (77.4%) achieved at least a 50% reduction in NRS pain scores. Clinically meaningful improvement was also observed in 83 of 84 patients (98.8%) for WOMAC scores and in 70 of 84 patients (83.3%) for both PCS-12 and MCS-12 scores.

**Figure 5:**
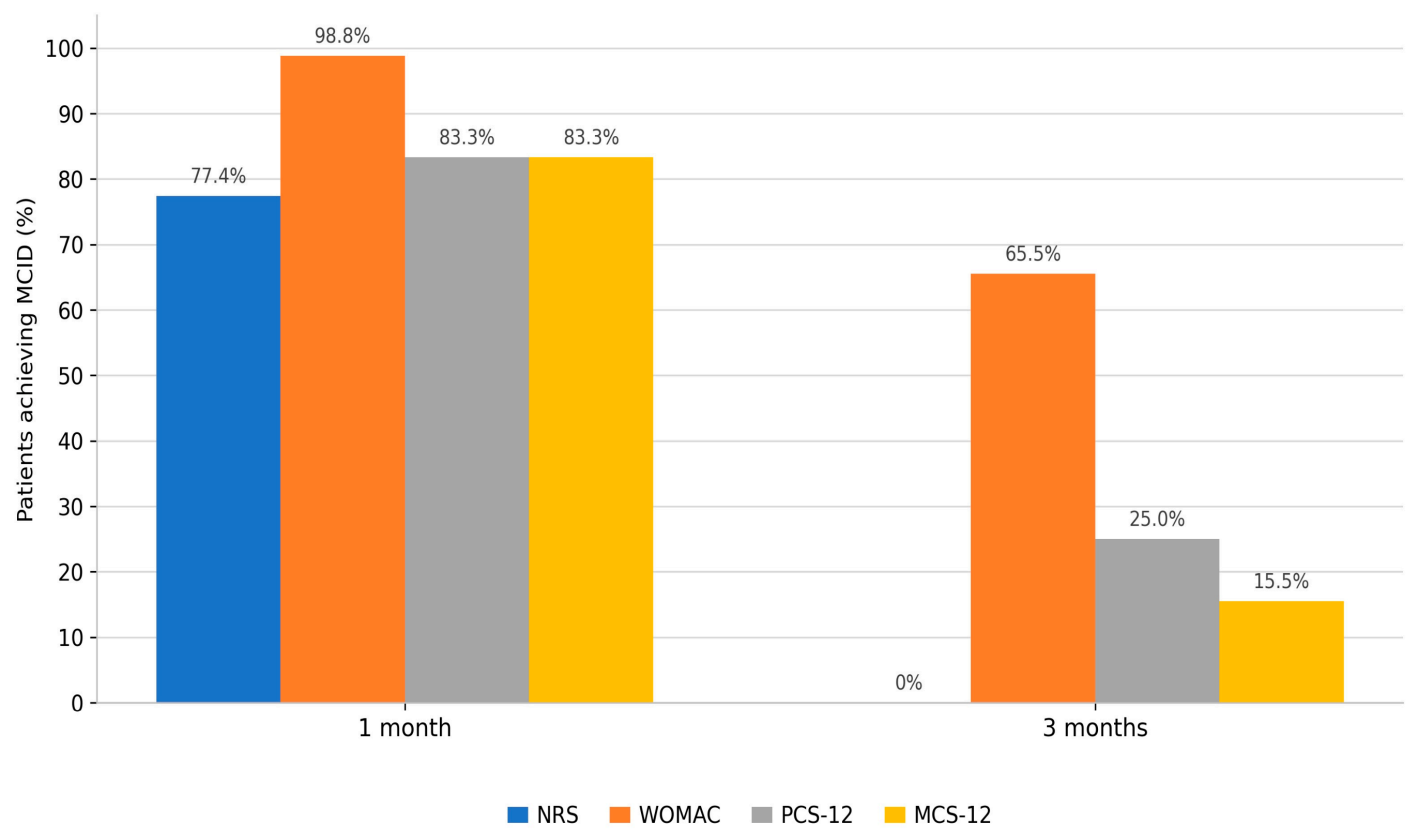
Proportion of patients achieving clinically meaningful improvement according to predefined MCID criteria. Values are presented as the percentage of patients meeting the predefined MCID criteria.

At the 3-month follow-up, clinically meaningful improvement persisted in 55 patients (65.5%) for WOMAC, 21 patients (25.0%) for PCS-12, and 13 patients (15.5%) for MCS-12. Although no patient continued to meet the predefined ≥50% NRS reduction criterion at 3rd month, responder rates for all outcomes remained significantly different between the 1- and 3-month assessments according to the McNemar test (all *p* < 0.001), indicating partial attenuation of treatment response over time (Table 4, Figure 5).

In the exploratory per-center analysis, improvements in NRS and WOMAC scores were not significantly different between centers after Holm adjustment. In contrast, improvements in PCS-12 and MCS-12 scores were significantly greater at Center 1 at both the 1- and 3-month assessments (Supplementary Table S1).

No serious procedure-related adverse events were recorded during the three-month follow-up period.

## 4. Discussion

In this prospective, multicenter, single-arm study, improvements in pain intensity, functional capacity, and health-related quality of life were observed following the IPACK block in patients with advanced knee osteoarthritis and predominant posterior knee pain. Although the magnitude of improvement was greatest at one month and attenuated by the third month, all outcome measures remained significantly different from baseline.

Importantly, the present study addresses a clinically underrecognized pain phenotype in knee osteoarthritis-predominant posterior knee pain. While most interventional strategies focus on anterior innervation, posterior capsular pain may significantly contribute to symptom burden, particularly in advanced disease. By specifically targeting this anatomical region, the IPACK block offers a mechanism-based approach that may explain the magnitude of clinical improvement observed in our cohort.

Existing literature on the use of IPACK for knee osteoarthritis is extremely limited [20]. The majority of studies focus on post-total knee arthroplasty, where IPACK has been shown to reduce posterior knee pain [21,22]. Our findings expand this knowledge by demonstrating that improvements in chronic posterior knee pain were observed following the IPACK block in osteoarthritis, not only limited to acute postoperative pain. The observed duration of pain relief is similar to other peripheral nerve blocks used in chronic knee pain, such as genicular nerve blocks, although it is shorter than the effects typically achieved with radiofrequency ablation [23,24]. Malhotra et al. compared two groups that experienced pain related to osteoarthritis, one receiving genicular nerve radiofrequency ablation and the other receiving a steroid-local anesthetic genicular nerve block. They reported that while both groups experienced pain relief, the block group showed a shorter duration of benefit, whereas the ablation group maintained significantly better NRS and WOMAC scores at 2 months, 3 months, 6 months, and 12 months [23]. In line with the generally time-limited effects of peripheral nerve blocks, the clinical benefit observed in our study was greatest at one month and diminished by three months, suggesting that the IPACK block may provide predominantly short-term pain relief. The mechanisms underlying an analgesic effect exceeding the known duration of neural blockade are unknown. This phenomenon may be explained by several mechanisms, including the fact that, while local anesthetics are known to have analgesic effects, they also have complicated anti-inflammatory actions, and they temporarily reduce nociceptive input [25,26].

Nonetheless, the temporal pattern of the observed response warrants particular attention. Improvements were greatest at the one-month assessment but had substantially attenuated by three months. The mean NRS score increased from 3.5 ± 1.5 at one month to 6.7 ± 1.3 at three months. Furthermore, although 65 patients (77.4%) achieved a reduction of at least 50% in NRS pain scores at one month, none continued to meet this responder criterion at three months. Similar attenuation was observed in functional and quality-of-life outcomes. Therefore, the findings primarily suggest a short-term improvement following a single IPACK block and do not demonstrate sustained clinically substantial pain relief at three months. Further controlled studies should evaluate the duration of the response and determine whether repeated applications provide additional or more sustained benefit.

In addition to the absence of randomized controlled trials about the IPACK block for knee osteoarthritis, the existing literature remains limited to case reports and studies in which IPACK is used as part of a combined intervention. Kim et al. described a 71-year-old patient with K-L grade 2 osteoarthritis complaining of right posterior knee pain who received an ultrasound-guided IPACK block and experienced marked improvement, with pain score decreasing from 6/10 to 1–2/10 and WOMAC score improving from 40 to 12 through one month and sustained benefit for five months [27]. Moreover, two recent studies further highlight the emerging interest in IPACK for knee osteoarthritis. A case description reported that combining a genicular nerve block with an IPACK block in a 71-year-old patient with bilateral K-L grade 2 osteoarthritis resulted in notable improvement in both anterior and posterior knee pain, suggesting that concomitant targeting of genicular nerves and the popliteal plexus may enhance overall analgesic response [28]. More importantly, a randomized study comparing adductor canal block (ACB) alone with combined ACB and IPACK block demonstrated that patients receiving the combined approach had significantly better pain reduction and functional outcomes across all follow-up intervals, underscoring the added value of posterior capsular analgesia [29]. Although these studies support the potential role of IPACK block in knee osteoarthritis, they either involve single-patient reports or evaluate IPACK block as part of a combined intervention. By contrast, the present study provides preliminary prospective data on clinical outcomes following an isolated IPACK block in patients with knee osteoarthritis and predominant posterior knee pain.

One possible explanation for the observed changes is the IPACK block’s targeted interruption of nociceptive input from the posterior knee capsule, innervated by articular branches of the tibial and obturator nerves [21,30]. Temporary reduction in nociceptive input may facilitate increased activity and improved biomechanics, potentially contributing to the observed functional improvements [31]. However, these mechanisms were not directly evaluated in the present study.

The IPACK block may occupy a complementary role within the current interventional treatment spectrum for knee osteoarthritis. While intra-articular injections primarily target synovial inflammation and provide variable short- to mid-term pain relief [32], and genicular nerve blocks or radiofrequency ablation (RFA) aim to modulate global knee joint nociception [33,34], the IPACK block specifically addresses posterior capsular innervation [35]. This is an often underrecognized source of pain in advanced disease. This targeted approach may explain its pronounced effect in patients with predominant posterior knee pain. Compared to genicular nerve interventions, IPACK is less likely to affect motor function and may offer a more focused analgesic profile, particularly for patients whose symptoms are not adequately controlled with conventional anterior-targeted strategies.

Pharmacological treatment remains an important component of real-world knee osteoarthritis management; nevertheless, persistent pain despite oral or topical analgesic therapy is frequently encountered in clinical practice, supporting the need to investigate additional treatment options for appropriately selected patients. These studies were included to provide context regarding commonly used noninterventional treatment options in knee osteoarthritis and to help position the potential role of IPACK within a broader multimodal treatment strategy. Two recent retrospective observational studies evaluated fixed-dose tramadol/paracetamol in patients with early-stage symptomatic knee osteoarthritis and reported improvements in pain and WOMAC scores during short- and mid-term follow-up [36,37]. However, both studies involved relatively small, uncontrolled populations with early-stage disease. Direct comparison with the present study is therefore not appropriate because of substantial differences in patient characteristics, osteoarthritis severity, treatment modality, and follow-up duration. Our findings do not indicate that the IPACK block is superior to pharmacological therapy or rehabilitation. Controlled comparative studies incorporating optimized pharmacological treatment and structured rehabilitation are needed to determine the potential role of the IPACK block within a multimodal treatment strategy.

Clinically, our findings suggest that the IPACK block may warrant further investigation as a motor-sparing analgesic approach for osteoarthritis patients with predominant posterior knee pain. It offers a valuable option for individuals who cannot tolerate NSAIDs, have contraindications to intra-articular steroid injections, or are unsuitable candidates for genicular nerve radiofrequency ablation. However, given its time-limited effect, a single IPACK injection should be incorporated into a broader multimodal pain management approach tailored to pain distribution rather than used as a sole long-term intervention.

On the other hand, total knee arthroplasty remains the definitive treatment for appropriately selected patients with advanced symptomatic knee osteoarthritis who have failed nonoperative management. The present findings should not be interpreted as supporting the IPACK block as a substitute for, or a means of delaying, indicated arthroplasty. Its potential role may instead be limited to short-term symptom management in selected patients, such as those awaiting surgery, those who are temporarily unsuitable for surgery because of comorbidities, or those who decline surgical treatment after informed discussion. Nevertheless, these possible applications were not directly evaluated in the present study. Comparative studies are required to determine whether the IPACK block provides meaningful clinical value when added to optimized conservative treatment or during the preoperative waiting period.

This study has several strengths. It includes a real-world patient population from two pain clinics, incorporates a relatively large sample size of 84 patients, and employs validated outcome measures such as NRS, WOMAC, and SF-12 scores to ensure a robust assessment of pain, functional status, and patient-reported outcomes. This study provides prospective clinical data on outcomes following an isolated IPACK block in patients with knee osteoarthritis and predominant posterior knee pain, an area in which the available literature remains limited. Unlike previous studies that primarily focused on postoperative settings or combined interventions, our findings provide preliminary observational data regarding posterior capsular analgesia in chronic osteoarthritis-related pain.

This study also has several limitations. First, the single-arm, nonrandomized design and absence of a comparator group limit causal inference and cannot ensure definitive therapeutic efficacy. Second, the three-month follow-up period allowed assessment only of short-term clinical changes and precluded evaluation of long-term durability. Moreover, because only a single IPACK injection was administered, the effectiveness, optimal timing, cumulative effects, and safety of repeated injections remain unknown. Third, potential confounding factors—including adherence to the exercise program, concomitant analgesic use, placebo and contextual effects, natural fluctuations in pain, and patient expectations—may have influenced the observed outcomes. Additionally, although patients were permitted to use NSAIDs or paracetamol as needed during follow-up, the type, dose, frequency, and duration of analgesic use were not systematically recorded. Consequently, the potential contribution of concomitant analgesic treatment to the observed improvements could not be evaluated, and the clinical changes cannot be attributed exclusively to the IPACK block. Although improvements were observed across multiple outcome measures, the absence of a control group prevents differentiation between changes associated with the IPACK block and those related to concomitant treatments, placebo and contextual effects, regression to the mean, or natural fluctuations in osteoarthritis-related pain. Fourth, neither participants nor outcome assessors were blinded to the intervention. The absence of blinded assessment and objective functional outcomes may therefore have influenced the magnitude of the observed changes. Fifth, outcomes were not analyzed separately according to Kellgren–Lawrence grade. Because the study was not designed or powered to compare grade III and grade IV subgroups, it remains unknown whether radiographic severity influenced the magnitude or duration of the observed changes. Additionally, the inclusion of predominantly advanced-stage osteoarthritis patients may limit generalizability. Furthermore, exploratory analyses identified between-center differences in the magnitude of improvement in PCS-12 and MCS-12 scores. Although procedural methods were standardized, unmeasured center-level differences in patient characteristics, clinical practice, rehabilitation adherence, or contextual factors may have contributed to this heterogeneity. Despite these limitations, the study provides important prospective clinical data addressing a relevant and underexplored clinical problem. Future research may include randomized controlled trials comparing the IPACK block with intra-articular injections, genicular nerve blocks, and conservative therapies. Studies are also needed to evaluate the effects of repeated IPACK sessions and to determine whether cumulative benefits occur over time. In addition, investigating the integration of IPACK with structured rehabilitation programs may clarify its role within multimodal treatment strategies. Finally, future work should assess long-term outcomes, including gait parameters and quality-of-life measures, to better define the procedure’s sustained clinical impact.

## 5. Conclusions

Enhancements in pain, functional capacity, and health-related quality of life were observed during the three-month period following a single ultrasound-guided IPACK block in patients with advanced knee osteoarthritis and predominant posterior knee pain. However, the absence of a control group precludes attribution of these changes exclusively to the intervention. These findings should therefore be considered preliminary and hypothesis-generating. Randomized controlled studies with larger samples, appropriate comparator groups, standardized monitoring of concomitant treatments, and longer follow-up periods are needed to clarify the effectiveness and safety of the IPACK block in this patient population.

## Figures and Tables

**Table 1:** Demographic data and patient characteristics of osteoarthritis.

Variable	Median (Min–Max)	Mean ± SD
Age (years)	64.0 (53.0–81.0)	64.5 ± 6.0
Body mass index (kg/m^2^)	30.1 (25.4–38.6)	30.5 ± 2.5
Duration of symptoms (months)	24.0 (6.0–72.0)	26.4 ± 16.6
Sex, *n* (%)		
Female	54 (64.3%)	
Male	30 (35.7%)	
Kellgren–Lawrence grade, *n* (%)		
Grade I	0 (0%)	
Grade II	0 (0%)	
Grade III	37 (44.0%)	
Grade IV	47 (56.0%)	

**Table 2:** Changes in clinical outcomes during follow-up and overall repeated-measures analysis.

Outcome	Baseline (Mean ± SD)	1st Month (Mean ± SD)	3rd Month (Mean ± SD)	*p*	χ^2^
NRS	7.8 ± 1.3	3.5 ± 1.5	6.7 ± 1.3	<0.001	154.943
WOMAC	71.5 ± 11.0	37.3 ± 10.4	60.2 ± 10.5	<0.001	159.958
PCS-12	31.8 ± 5.4	39.7 ± 5.5	35.6 ± 5.3	<0.001	146.881
MCS-12	34.0 ± 4.3	41.5 ± 4.9	37.7 ± 4.7	<0.001	151.922

Values are presented as mean ± standard deviation for descriptive purposes. Overall comparisons across the three assessment time points were performed using the Friedman test, followed by Bonferroni-adjusted Wilcoxon signed-rank tests for pairwise comparisons.

**Table 3:** Magnitude of within-patient changes from baseline at the 1- and 3-month follow-up assessments.

Outcome	Comparison	Mean Difference (95% CI)	Z	*p*	Effect Size (r)
NRS	Baseline vs. 1st month	4.21 (3.96–4.47)	−7.985	<0.001	0.87 (Large)
NRS	Baseline vs. 3rd month	1.06 (0.88–1.24)	−7.080	<0.001	0.77 (Large)
WOMAC	Baseline vs. 1st month	34.17 (31.99–36.34)	−7.963	<0.001	0.87 (Large)
WOMAC	Baseline vs. 3rd month	11.25 (9.95–12.55)	−7.814	<0.001	0.85 (Large)
PCS-12	Baseline vs. 1st month	7.84 (7.17–8.50)	−7.947	<0.001	0.87 (Large)
PCS-12	Baseline vs. 3rd month	3.71 (3.31–4.12)	−7.934	<0.001	0.87 (Large)
MCS-12	Baseline vs. 1st month	7.50 (6.93–8.07)	−7.956	<0.001	0.87 (Large)
MCS-12	Baseline vs. 3rd month	3.56 (3.18–3.94)	−7.891	<0.001	0.86 (Large)

Effect sizes were calculated as r = Z/√N and interpreted according to Cohen’s criteria.

**Table 4:** Patients achieving clinically meaningful improvement according to predefined MCID criteria.

Outcome	1st Month *n* (%)	3rd Month *n* (%)	χ^2^	*p*
NRS (≥50% reduction)	65 (77.4)	0 (0.0)	63.015	<0.001
WOMAC (≥10-point reduction)	83 (98.8)	55 (65.5)	26.036	<0.001
PCS-12 (≥5-point increase)	70 (83.3)	21 (25.0)	45.176	<0.001
MCS-12 (≥5-point increase)	70 (83.3)	13 (15.5)	53.153	<0.001

McNemar’s test was used to compare responder rates between the 1- and 3-month follow-up visits.

## Data Availability

The data that support the findings of this study are available from the corresponding author upon reasonable request.

## Supplementary Materials

The following supporting information can be downloaded at https://www.mdpi.com/article/10.3390/jcm15166410/s1. Table S1: Exploratory Per-Center Comparison of Clinical Outcomes.

## Abbreviations

The following abbreviations are used in this manuscript:

ACB	Adductor canal block
CI	Confidence interval
IA	Intra-articular
IPACK	Interspace between the popliteal artery and capsule of the posterior knee
IV	Intravenous
KL	Kellgren–Lawrence classification of osteoarthritis
KOA	Knee osteoarthritis
LA	Local anesthetic
MCID	Minimal clinically important difference
MCS-12	Mental Component Score of the Short Form-12 Health Survey
NRS	Numeric Rating Scale
NSAIDs	Nonsteroidal anti-inflammatory drugs
PA	Popliteal artery
PCS-12	Physical Component Score of the Short Form-12 Health Survey
PV	Popliteal vein
RFA	Radiofrequency ablation
US	Ultrasound
WOMAC	Western Ontario and McMaster Universities Arthritis Index
