# Supplementary material for: Clinical Outcomes of Ultrasound-Guided IPACK Block for Posterior Knee Pain in Advanced Osteoarthritis: A Multicenter Prospective Single-Arm Study
Source: J Clin Med. 2026 Aug 19;15(16):6410. doi: 10.3390/jcm15166410 (PMC13513714; doi:10.3390/jcm15166410)
Supplement: Supplementary file 1 [file jcm-15-06410-s001.zip › jcm-4463423-supplementary.pdf]

Supplementary Table S1. Exploratory Per-Center Comparison of Clinical Outcomes

Center 1: participants 1–44 (n=44); Center 2: participants 45–84 (n=40)

Panel A. Outcome scores at each assessment point

Outcome	Assessment	Center 1 (n=44)	Center 2 (n=40)	Unadjusted p
NRS	Baseline	7.70 ± 1.23	7.80 ± 1.36	0.775
NRS	1 month	3.25 ± 1.45	3.85 ± 1.49	0.011
NRS	3 months	6.45 ± 1.37	6.95 ± 1.20	0.063
WOMAC	Baseline	71.18 ± 12.25	71.80 ± 9.68	0.946
WOMAC	1 month	37.09 ± 10.52	37.55 ± 10.40	0.700
WOMAC	3 months	59.95 ± 11.21	60.52 ± 9.90	0.897
PCS-12	Baseline	30.70 ± 5.27	33.09 ± 5.37	0.034
PCS-12	1 month	39.52 ± 4.89	39.84 ± 6.23	0.540
PCS-12	3 months	35.17 ± 4.91	35.97 ± 5.76	0.402
MCS-12	Baseline	33.88 ± 3.79	34.22 ± 4.84	0.606
MCS-12	1 month	42.20 ± 4.37	40.81 ± 5.35	0.336
MCS-12	3 months	38.07 ± 4.34	37.08 ± 5.09	0.632

Panel B. Between-center comparison of improvement from baseline

Outcome	Comparison	Center 1 improvement	Center 2 improvement	Unadjusted p	Holm-adjusted p
NRS	Baseline to 1 month	4.45 ± 1.19	3.95 ± 1.15	0.018	0.067
NRS	Baseline to 3 months	1.25 ± 0.87	0.85 ± 0.74	0.017	0.067
WOMAC	Baseline to 1 month	34.09 ± 10.62	34.25 ± 9.48	0.657	1.000
WOMAC	Baseline to 3 months	11.23 ± 4.94	11.28 ± 7.06	0.918	1.000
PCS-12	Baseline to 1 month	8.82 ± 2.95	6.75 ± 2.88	<0.001	0.004
PCS-12	Baseline to 3 months	4.46 ± 1.72	2.89 ± 1.71	<0.001	<0.001
MCS-12	Baseline to 1 month	8.32 ± 2.52	6.59 ± 2.46	0.002	0.012
MCS-12	Baseline to 3 months	4.20 ± 1.68	2.86 ± 1.54	<0.001	0.002

Notes. Values are mean ± standard deviation for descriptive consistency with the main manuscript. Between-center comparisons were performed using the Mann–Whitney U test. Improvement was defined so that positive values indicate clinical improvement (decreases for NRS and WOMAC; increases for PCS-12 and MCS-12). Holm adjustment was applied across the eight change-score comparisons in Panel B. The analyses were exploratory. NRS, Numeric Rating Scale; WOMAC, Western Ontario and McMaster Universities Osteoarthritis Index; PCS-12, Physical Component Summary; MCS-12, Mental Component Summary.
